# Psychometric properties of trunk impairment scale in children with spastic diplegia

**DOI:** 10.1038/s41598-021-98104-7

**Published:** 2021-09-17

**Authors:** Vedasri Dasoju, Rakesh Krishna Kovela, Jaya Shanker Tedla, Devika Rani Sangadala, Ravi Shankar Reddy

**Affiliations:** 1 Department of Physical Therapy, Krishna Institute of Medical Sciences, Secunderabad, India; 2 Department of Neuro Physiotherapy, Ravi Nair Physiotherapy College, Datta Meghe Institute of Medical Sciences, Sawangi (Meghe), Wardha, Maharashtra India; 3 Department of Medical Rehabilitation Sciences, College of Applied Medical Sciences, King Khalid University, Abha, Kingdom of Saudi Arabia

**Keywords:** Neuroscience, Medical research

## Abstract

The Trunk Impairment Scale (TIS) is a valid and reliable tool to assess trunk impairment in children with heterogeneous cerebral palsy. The purpose of this study was to determine the reliability and validity of the TIS in assessing children with spastic diplegic cerebral palsy. The sample was a total of 30 subjects (15 = boys, 15 = girls). All subjects underwent an assessment of the sitting component of the Gross Motor Function Measure-88 and TIS by rater 1. Rater one observed video recordings within 24 h and scored TIS for intra-rater reliability, while rater two did likewise after 48 h for inter-rater reliability. The mean and standard deviation of the TIS and sitting components of the Gross Motor Function Measure-88 were 15.66 ± 4.20 and 52.36 ± 6.26, respectively. We established intra-rater and inter-rater reliability of the TIS with Intra Class Correlation Coefficient 0.991 and 0.972, respectively. The concurrent validity of the TIS with the sitting component of the Gross Motor Function Measure-88 was good, with an r-value of 0.844 (*p* < 0.001). This study showed the excellent intra-rater and inter-rater reliability and high concurrent validity of the TIS in assessing children with spastic diplegic cerebral palsy.

## Introduction

Trunk control is defined as part of postural control. The ability to control the torso and perform selective movements of the trunk^1^, it is a critical element of maintaining postural control and establishing balance reactions. It acts as a stable base for the limbs' movement, thereby enabling the functions of the upper and lower extremities^2,3^. Motor development is defined as the changes that occur in motor skill behavior over time^4^. Postural control development is a complex process that occurs phylogenetically with the cephalocaudal direction, which starts in early life^5^.

During the developmental process of prone weight bearing through the forearms and hands, the shoulder girdle and upper thoracic muscular control facilitate the head's stabilization over the upper trunk. The lower trunk development occurs when sitting with propped arms to sitting without support due to control in the pelvic and lower limb muscles. Both upper and lower trunk control enable selective head and limb movements and anticipatory postural adjustments^6^. Cerebral palsy (CP) is a group of neurodevelopmental disorders of posture and movement due to non-progressive lesions in the developing fetal or infant brain. This motor disorder is characterized by neuromuscular dysfunction and impairments in sensation, perception, communication, and cognition and can be associated with intellectual disability and seizures. These impairments contribute to activity limitations and participation restrictions^7^.

Cerebral palsy is classified according to Surveillance of Cerebral Palsy in Europe as bilateral spasticity, unilateral spasticity, dyskinesia, and ataxia^8^. The neuromuscular impairments in children with CP are abnormal tone, weakness, and poor trunk control. Trunk control impairment plays a central role in motor dysfunction in children with CP^2^. Insufficient trunk postural control is a severe concern in children with CP as it causes inadequate postural control and trunk stability. To maintain stability, children with CP use their limbs not free to use limbs for the normal purpose. Thereby, these children demonstrate difficulties maintaining sitting positions, playing in sitting positions, and performing functional hand movements such as eating^9^. Studies have reported that children with CP demonstrate trunk impairment difficulties in sitting and standing skills and functions such as reaching and walking^10^. In spastic diplegia, the upper extremities are less involved than the lower extremities, and the trunk is often involved^7^. This trunk instability contributes to difficulties in postural muscle activation in task-specific conditions^11^. Yildiz et al. stated that bilateral CP demonstrated insufficient trunk control and reduced upper extremity functions such as grip, protective extension, and weight-bearing compared to unilateral CP^12^. Trunk control abnormalities are the major limitation to motor development in children with spastic diplegia. Trunk instability is evident in spastic diplegic CP observed by oscillations in the center of pressure, altering the balance and further delaying gross motor function. The delay in gross motor function and altered balance lead to difficulty in performing activities of daily living and poor walking abilities^13^.

Due to the importance of trunk control, trunk impairment needs to be evaluated objectively in clinical practice. In the literature, there are many clinical outcome measures, such as Seated Postural Control Measure(SPCM)^14,18^, Segmental Assessment of Trunk Control (SATCo)^15,18^, Trunk Control Measurement Scale (TCMS)^9,18^, Trunk Impairment Scale (TIS)^16,18^ and Spinal Alignment and Range of Motion Measure (SAROMM)^17,18^. SPCM has poor reliability; SAROMM provides information only on postural characteristics of the trunk, not on the trunk control in static and dynamic trunk control. SATCO also evaluates static trunk only; it does not contain the items for evaluating dynamic trunk control^9^. TCMS and TIS measure both static and dynamic trunk control^9^. TCMS is more time-consuming, while TIS is less time-consuming^19^. In TCMS on selective movement control, children can perform some components rotation of upper trunk and rotation of lower trunk unilaterally. In TIS, children can perform the coordination components like the rotation of the upper trunk and rotation of the lower trunk bilaterally. As in children with SDCP, trunk dissociation is important to evaluate bilaterally; we feel that TIS is superior to TCMS in assessing trunk impairment in these children. Moreover, TCMS consumes more time and is difficult to administer than TIS. In recent studies showed that TIS dynamic sitting balance items were positively correlated with trunk control during gait in children with cerebral palsy^19^. Since TIS is well-established and highly associated with the improvement needs of Physical Therapists, our interest was to examine its use further.

The TIS, developed by Verheyden et al., is a valid and reliable tool for evaluating trunk motor impairment in stroke subjects^20^. It assesses static sitting and dynamic sitting balance and trunk coordination, and several studies have used it to assess trunk impairment in subjects who have had strokes^21^, traumatic brain injuries^22^, who have multiple sclerosis^23^, and early-stage Parkinson's disease^24^.

Saether administered the TIS to assess children and adolescents with CP and proved its reliability and validity^16^. In the literature, many authors have conducted studies to establish TIS's performance in assessing children and adolescents with CP and to estimate the TIS's validity and responsiveness^25,26^. Many studies proved TIS validity with the Gross Motor Function Measure-88 (GMFM-88); it contains 88 items categorized into five dimensions: lying and rolling, sitting, crawling and kneeling, standing and walking, jumping, and running. The sitting dimension of the GMFM-88 consists of 20 items like transitions from supine to rolling, pull to sit, sitting on different surfaces such as mat, floor, small and large benches with support and without the support, which are scored on an ordinal scale. This outcome measure quantitatively evaluates motor functions, and many studies have used GMFM-88 to assess the effectiveness of interventions in children with CP^27^.

However, these studies included heterogeneous groups of CP children. Spastic diplegic CP (SDCP) is one of the commonest forms of cerebral palsy^28^, where trunk control plays a crucial role in determining functional and gait capabilities. The deviations during gait in the trunk can be observed from the primary impairment in the trunk and the impairments in lower limbs. Moreover, primary impairments in the trunk produce compensatory movements in the lower extremities, for example, by allowing the pelvis to rotate anteriorly and leading to increased hip flexion^19^. Saether et al. established a good correlation of trunk function measured by TIS and TCMS during gait. However, TIS- DSB subscale is easy to administer, less time-consuming and demonstrated the highest correlation with gait parameters^19^. Hence this study focuses on the TIS's reliability and validity while assessing children with spastic diplegic CP.

## Methodology

After obtaining the King Khalid University research ethics committee approval (ECM#2019-227-HAPO-06-B-001), all the experiments were performed in accordance with relevant guidelines and regulations of the research ethics committee King Khalid University. In this cross-sectional study, we recruited, based on medical records, a total of 30 children with SDCP between the ages of three to 10 years of both genders from the authors’ university outpatient Physical Therapy clinic. We calculated the sample size using ClinCalc statistical software(version-©2021 - ClinCalc LLC,www.clincalc.com)^29^ for the following estimates^16^ the mean population was 5, and the mean study group was 3.87, alpha was 0.05, beta 0.2, and power 0.8. Both the tests TIS and siting dimension of GMFM-88 requires children to sit independently for application. Therefore, children with SDCP who could sit without support for 10 s on a bench and follow instructions were included. This included children with SDCP from level I to level IV, according to the Gross Motor Function Classification System^26,30^. Children with SDCP who had undergone surgery within the preceding six months, or had fixed deformities, intellectual disabilities, or seizures, or were taking anti-epileptic medications were excluded from the study.

The TIS evaluates trunk impairment according to three subcomponents: static sitting balance (TIS-SSB), dynamic sitting balance (TIS-DSB), and trunk coordination (TIS-C). The TIS-SSB contains three items, in which the first and second items are scored on zero to two points, whereas the third item is scored on zero to three on an ordinal scale. The maximum score of this subscale is seven. The TIS-DSB consists of ten items, all items scored on zero to one point on an ordinal scale. This subscale's maximum score is ten. The TIS-C has four items in which the first and third items were scored on zero to two on an ordinal scale, and the second and fourth are scored on zero or one point. The maximum score of this subscale is 6. The TIS contains a total of 17 items, and the three subtotal scores range from 0 (poor performance) to 23 (best performance)^25^. Saether et al. modified the TIS in terms of items 8 and 10 of the dynamic balance subcomponents; they considered whether the feet lose contact with the floor, not the heel, as spastic diplegic children have an equinus position. Children were given physical guidance if required to help them understand the task^16^.

### Procedure

The authors explained the purpose of the study and the test's application to parents interested in allowing their children with SDCP to participate in the study. We obtained a written consent form from the parents of the eligible children. For the TIS application, the children sat on a wide bench, with thighs horizontal, feet flat (if possible) and resting supported, knees flexed to 90 degrees, no back support, and hands and forearms resting on the thighs. For children with SDCP, the starting position was with the arms in a neutral position. They were permitted to wear minimal clothing and regular footwear (orthoses, shoes). For item three in the static sitting balance component, a red mark was placed on the bench 10 cm from the child's pelvis's rear to make observations of trunk movement more than 10 cm backward easier. Two raters having ten years of experience in pediatric physical therapy participated in data collection. Rater one assessed three subcomponents of the TIS, with three trials for each item, and the best performance score was used for data analysis. If required, physical guidance was provided by the rater during the application of the test^16^. The application of the TIS was video recorded for all the children. This video recording was observed and scored by rater one for intra-rater reliability within 24 h intervals. After 48 h, rater two watched the video recordings and scored the TIS for inter-rater reliability. After applying the TIS, a significant rest period was given, followed by an assessment of the Gross Motor Function Measure-88 (GMFM) sitting component that was done for all 30 children by rater one. Gross Motor Function Measure-88 is a reliable and valid tool to measure functional performance in children with CP^27^. We considered the total score of sitting dimensions to calculate the concurrent validity. Based on the children's performance, apparent differences were noticed in their performance in some age groups. We further divided the children into three age groups, 3–5, 6–7, and 8–10 years, and also analyzed their data to provide more insight into the TIS and GMFM performance.

### Statistical analysis

Statistical Package for Social Sciences (SPSS) version 22 was used for data analysis, and univariate analysis of the children's demographic characteristics and their test scores was performed using descriptive statistics. The Shapiro–Wilk test was used to find the normal distribution of the variables, and the data were distributed normally and represented in mean and standard deviations. Similarly, we assessed the inter- and intra-rater reliability of the TIS by using intra-class correlations (ICC). Moreover, we also computed the standard error of measurement (SEM) for representing absolute reliability. We calculated the SEM as the square root of the mean within-subject variance. The measurement error was expressed according to the SEM, and when there are lower SEM values, then there will usually be less measurement error. Subsequently, we determined the smallest detectable change for the two measurement differences of the same subject. In 95% of the pairs of observations, we anticipated the slightest noticeable change (SDD) to be less than √2 × 1.96 × SEM = 2.77 SEM.20.

For intra rater reliability, we used ICC of two-way random effects, single measurement with consistency. For assessing inter-rater reliability, we used a mixed-effects model and the mean of multiple raters with consistency. We further used a Bland Altman Graph for comparing the two raters' measurements. The graph plots the differences between two raters against their mean values. We needed to create two new variables based on the two rater values for plotting the Bland Altman graph. The first variable is the difference between rater one and rater two values of TIS. The second variable is the mean of rater one and rater two values of TIS. By applying a one-sample t-test for the difference variable, we obtained mean, standard deviation, and *p* values. Using this mean and standard deviation, we further calculated the upper and lower 95% confidence intervals. Using scatter dot graphs in SPSS, we obtained a Bland Altman graph with the Y-axis representing differences between the two raters and the X-axis representing the mean value of the two raters' measurements.

We assessed concurrent validity using the Pearson correlation coefficient. A *p* value of less than 0.05 was considered significant with 95% confidence intervals. For interpreting ICC, we followed the classification provided by Koo and Li ^31^ and, for interpreting the Pearson correlation r-value, we followed the classification described by Schober and Schwarte ^32^.

### Ethics approval

This study obtained ethical approval from the research ethics committee of the University.

### Consent to participate

Authors obtained the consent from the parents of the children who participated in the study.

### Consent for publication

All the authors permit to publish this article in Scientific Reports.

## Results

We studied 30 children (15 boys and 15 girls) with SDCP. Mean ± SD for their age was 6.03 ± 2.51 yrs. The Mean ± SD of their TIS and sitting component GMFM-88 scores were 15.66 ± 4.2 and 52.36 ± 06.26, respectively. Moreover, the SEM values for the TIS scores of raters one and two were 0.767 and 0.726. The SEM and SDD for the difference between rater one and rater two were 0.21 and 0.58, respectively. The intra- and inter-rater reliability ICC values were excellent, and the concurrent validity r value was outstanding. All these variables are shown in Table 1.

**Table 1:** Representing age of the subjects, GMFCS level, TIS values, sitting dimension GMFM-88 scores, validity, and reliability values of the TIS scale.

Variable	Values
Age (years) (Mean ± SD)	6.03 ± 2.5
Distribution of children as per GMFCS E&R (N)	GMFCS-I – 3
	GMFCS-II – 12
	GMFCS-III – 11
	GMFCS-IV – 4
TIS score (Mean ± SD)	15.66 ± 4.20
Sitting dimension GMFM-88 score (Mean ± SD)	52.36 ± 6.26
Intra-rater reliability (ICC)	0.991
Inter-rater reliability (ICC)	0.972
Concurrent validity (r-value)	0.844 (*p* < 0.001)

SD: Standard Deviation, GMFCS E&R: Gross Motor Classification System Expanded & Revised, TIS: Trunk Impairment Scale. GMFM: Gross Motor Function Measure, ICC: Intraclass Correlation Coefficient, N: Number of subjects.

**Figure 1:**
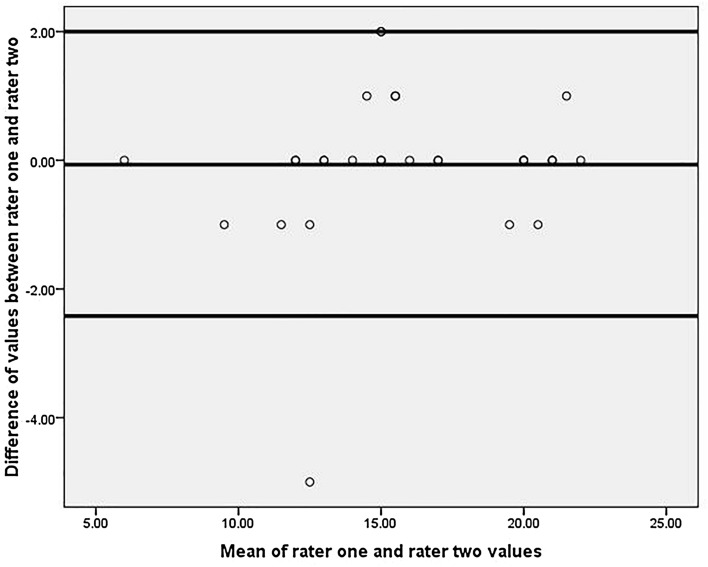
Bland Altman Graph representing inter-rater reliability. The horizontal middle dark line represents the mean, and the upper and lower dark line represents higher and lower standard deviations.

**Figure 2:**
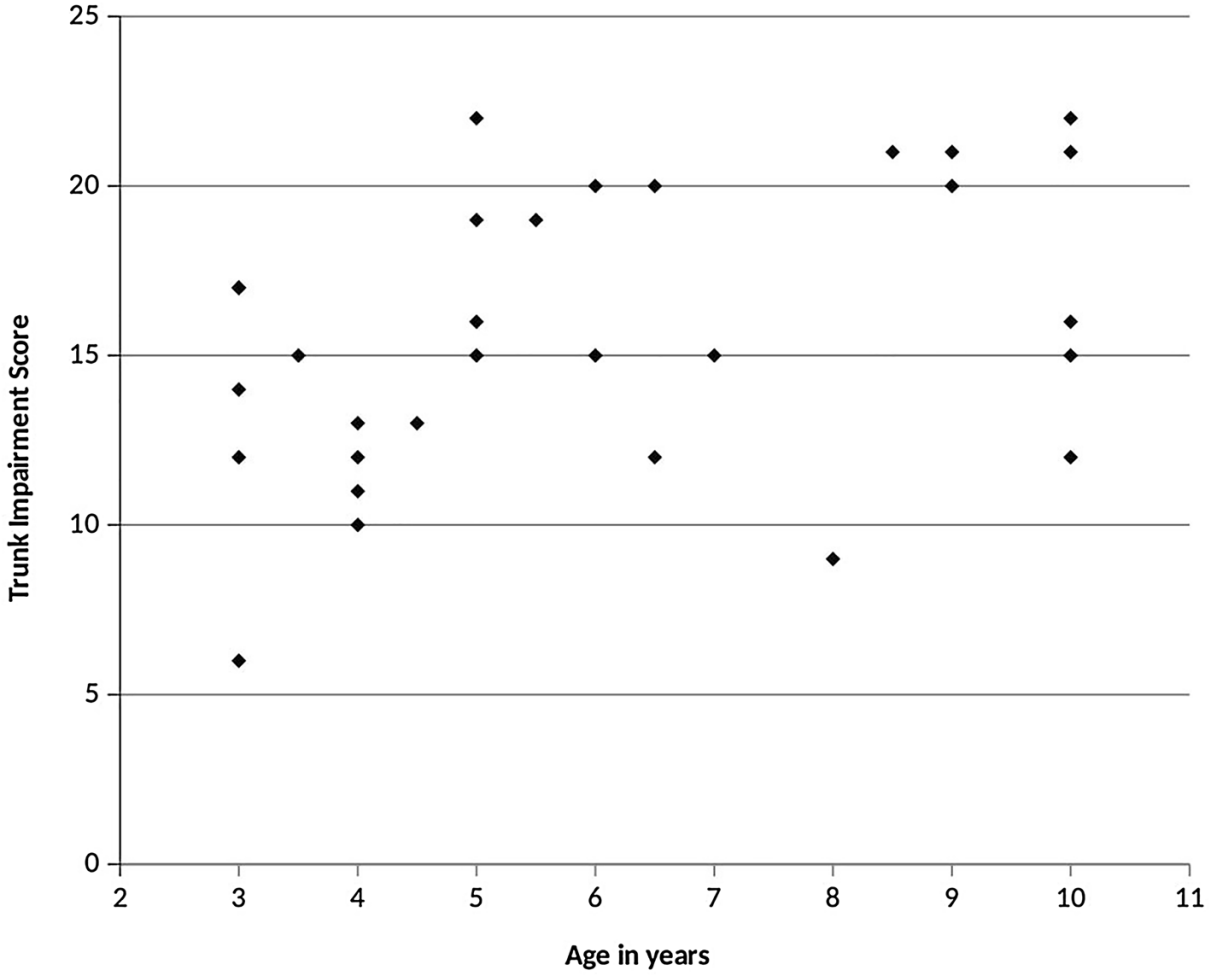
Scores trunk impairment scale based on the children’s age.

Based on the children's TIS performance, they were further divided into three age groups, 3–5, 6–7, and 8–10 years. The Mean ± SD for the age of 3–5-years group was 3.9 ± 0.8 years, for the age group of 6–7 years, it was 6.2 ± 0.5 years, and for the age group of 8–10 years, it was 9.3 ± 0.7 years. The Mean ± SD of their TIS and sitting component GMFM-88 scores according to age groups are demonstrated in Table 2. The intervals of agreement between the two raters were described by using Bland Altman Graph. The X-axis represented the mean values of rater one and two, while Y-axis represented the difference between rater one and rater two. The inter-rater reliability was plotted with the Bland Altman Graph, as shown in Fig. 1. Further, the TIS scores based on the children's age were plotted on the graph in Fig. 2.

**Table 2:** TIS values, sitting dimension GMFM-88 scores according to age groups.

Age group	Number	Mean age (Mean ± SD)	TIS Intra-rater scores (Mean ± SD)		TIS rater 2 scores (Mean ± SD)	GMFM-88 sitting dimension score (Mean ± SD)
			1st time	2nd time		
3–5 years	15	3.9 ± 0.8	14.13 ± 3.88	14.6 ± 3.99	14.8 ± 3.80	51 ± 6.55
6–7 years	6	6.2 ± 0.5	16.83 ± 3.31	16.83 ± 3.31	16.83 ± 3.6	55.33 ± 2.58
8–10 years	9	9.3 ± 0.7	17.44 ± 4.66	17.44 ± 4.66	17.22 ± 4.54	52.66 ± 7.24

TIS: Trunk Impairment Scale, SD: Standard Deviation, GMFM: Gross Motor Function Measure.

## Discussion

This study proved excellent intra- and inter-rater reliability, with ICC values of 0.991 and 0.972, respectively. These findings are in accordance with the study conducted by Seather et al. on children with CP between 5 and 12 years old, in which they found high intra- and inter-rater reliability with ICC values of 0.94 and 1^33^. An explanation for this is that, in our study, both raters observed and scored the children's performance of the TIS after analyzing the video recordings, as did the raters in Seather et al.'s study. A further 2013 study by Seather et al. on children with CP between 3 and 19 years old also demonstrated high inter and intra-rater reliability, with ICC values of 0.82 and 0.98, respectively. In this study, raters also analyzed video recordings and scored the children's performance on the TIS on two occasions^16^. If the test is applied by a rater who is unknown, which makes the child insecure and frightened, thereby reduces the child's performance. Thus video recording is a useful tool to create a qualitative and optimal testing situation. Further, video scoring can allow for blinded evaluation. It may also increase reliability by enabling the raters to score by pausing the video and reviewing items in case of doubt^34^. Hence, as mentioned above, we believe that the advantages of video recordings could have contributed to high intra and inter-rater reliability.

Pavao et al. conducted a study on children with CP to determine the TIS's discriminant ability and criterion validity and found an excellent correlation of 0.944 (*p* < 0.001) between the TIS and the GMFM^26^. TIS assesses the primary impairment of trunk control. GMFM assesses the gross motor function; the scoring for each component of the TIS scale is well elaborated based on the child's performance, and TIS does not require much equipment compared to GMFM. TIS evaluates children's performance without disturbing the positions. Thereby: in clinical practice, TIS can be recommended to assess trunk impairment in children. Saether et al. conducted a study to determine the concurrent validity of the TIS with the GMFM and found a high correlation between the TIS score and dimensions of the GMFM, with Spearman’s rho − 0.80 to 0.87^16^. These findings suggest that TIS is a valid measure to assess trunk control and determine motor impairment in children with CP because TIS evaluates the static, dynamic, and coordination components of trunk control using less equipment and consumes less time for application of the test.

The TIS's concurrent validity with the sitting component of the GMFM-88 was proved, with a Pearson correlation coefficient value of 0.844. This study also demonstrated that TIS is a valid measure to assess trunk control in children with SDCP.

A study conducted by Heyrrman et al. to evaluate the psychometric properties of the TCMS in children with spastic CP proved the intra-rater reliability and test-rest reliability with ICC values of 0.91 to 0.99. Construct validity of TCMS with GMFM had a Spearman rank correlation of 0.88^9^. Our study findings also proved the intra- rater and inter-rater reliability of TIS with ICC values of 0.991 and 0.972, respectively. Concurrent validity of the TIS with the GMFM-88 had a Pearson correlation coefficient of 0.88 in children with spastic diplegia. This suggests that TIS is also a valid and reliable measure for assessing trunk control in children with spastic diplegia^33^. TIS evaluates trunk control in static sitting balance, dynamic sitting balance, and coordination components on an ordinal scale. Sitting dimensions of GMFM-88 assess the gross motor function like maintaining the static sitting posture with support or without support on different surfaces, and a dynamic sitting balance scored on an ordinal scale. Some of the TIS scale components and GMFM-88 are similar, for example, maintaining a sitting position for 10 s on a bench, Sitting on a mat, and touching the toy right side and left side, which is almost similar to rotating the upper trunk. These explanations can be reasons for that TIS correlated well with the sitting dimension of GMFM-88.

In our study, total sores of TIS are higher as the age increases. This finding is consistent with the research conducted by Sæther and Jorgensen ^33^. This could be attributed to bigger age group children might have shown good capabilities to understand the instructions, so better performance on TIS scores. In contrast, smaller age groups showed less capacity to understand instructions and less performance on TIS scores.

This study's limitations were that only children aged 3–10 were included and no other age groups. Subgroup analysis based on the GMFCS level was also not performed due to the small sample in each level of GMFCS. In the current study, we feel that the child's capabilities represent the extent to which they comprehend the instructions. In our research, objective measurement of the spasticity was not performed, and 2D video recordings assessed reliability. Due to lack of availability, more sensitive and precise methods, such as the center of pressure displacement by a force plate, were not considered. Future studies should consider including larger sample sizes in each level of GMFCS, covering all the age groups, including the gold standard method of the center of pressure displacement by force plate or any other better measures to prove concurrent validity. Further studies should also include 3D video recording for analyzing the reliability, measure the spasticity by objective methods to find the association of spasticity on trunk control in SDCP.

## Conclusion

This study showed that the TIS has excellent inter and intra-rater reliability in children with SDCP. In addition, it proved the concurrent validity of the TIS with the sitting component of the GMFM88 to be high. Therefore, the TIS is a reliable and valid measure to assess trunk control in children with SDCP.

## Data Availability

The data and material of the current study will be available from the corresponding author on reasonable request.
