# Author Correction: Psychometric properties of trunk impairment scale in children with spastic diplegia

**DOI:** 10.1038/s41598-021-99453-z

**Published:** 2021-10-07

**Authors:** Vedasri Dasoju, Rakesh Krishna Kovela, Jaya Shanker Tedla, Devika Rani Sangadala, Ravi Shankar Reddy

**Affiliations:** 1 Department of Physical Therapy, Krishna Institute of Medical Sciences, Secunderabad, India; 2 Department of Neuro Physiotherapy, Ravi Nair Physiotherapy College, Datta Meghe Institute of Medical Sciences, Sawangi (Meghe), Wardha, Maharashtra India; 3 Department of Medical Rehabilitation Sciences, College of Applied Medical Sciences, King Khalid University, Abha, Kingdom of Saudi Arabia

Correction to: *Scientific Reports* 10.1038/s41598-021-98104-7, published online 17 September 2021

The original version of this Article contained an error in Affiliation 2, which was incorrectly given as “Department of Neuro Physiotherapy, Datta Meghe Institute of Medical Sciences, Sawagni (Meghe), Wardha, Maharashtra, India”.

The correct affiliation is listed below:

Department of Neuro Physiotherapy, Ravi Nair Physiotherapy College, Datta Meghe Institute of Medical Sciences, Sawangi (Meghe), Wardha, Maharashtra, India.

The original Article has been corrected.

